# Single-Electron Charging of Thioctic Acid Monolayer-Protected Gold Clusters

**DOI:** 10.1021/acs.jpclett.2c03940

**Published:** 2023-02-03

**Authors:** Jose M. Abad, Marcos Pita, Antonio L. De Lacey

**Affiliations:** Instituto de Catálisis y Petroleoquímica, CSIC. C/Marie Curie 2, 28049Madrid, Spain

## Abstract

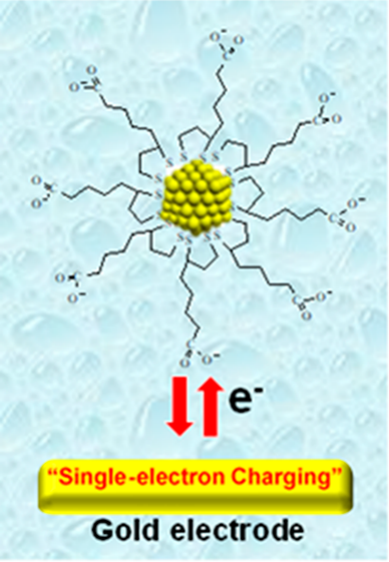

There is great interest in the use of Monolayer-Protected Gold Clusters (AuMPCs) as nanoscale capacitors in aqueous media for nanobiotechnological applications, such as bioelectrocatalysts, biofuel cells, and biosensors. However, AuMPCs exhibiting subattofarad double-layer capacitance at room temperature, and the resolution of single-electron charging, has been mainly obtained in an organic medium with nonfunctional capping ligands. We report here the synthesis of Thioctic Acid Monolayer-Protected Au Clusters (TA-AuMPCs) showing electrochemical single electron quantized capacitance charging in organic and aqueous solutions and when immobilized onto different self-assembled monolayer-modified gold electrodes. The presence of functional carboxylic groups opens a simple strategy for interfacing a nanoparticle assembly to biomolecules for their use as electron donors or acceptors in biological electron transfer reactions.

Monolayer-protected gold nanoclusters (AuMPCs) have received extensive attention as a hot topic for applications in different fields due to their unique electronic and chemical properties.^1−7^ They are originated from quantum effects inherent to discrete electron energy states, which arise from gold core dimensions on the nanometer scale.^8−11^ Sequential single-electron transfer events for both freely diffusing and electrode-attached MPCs can be observed at room temperature similar to either the “Coulomb Staircase” behavior or to sequential electrochemical redox reactions.^11−28^ MPCs can act as subattoFarad (aF) molecular capacitors resulting from a combination of their small metallic core size and the dielectric properties of the organic protecting layer. Successive one-electron charging steps of the MPC cores, termed quantized double layer charging (QDL), can be observed with potential spacing between these charging events given by Δ*V* = *e*/*C*_MPC_ (*C*_MPC_ = capacitance value) that are significantly larger than room temperature thermal energy (*kBT*/*e*), thus allowing their use as electron donors and acceptors in electron transfer reactions.^12,16,21,25−28^ This phenomenon has been observed electrochemically mainly in organic media for hydrophobic alkanethiolate- and arene thiolate-MPCs with a monodisperse core size.^13,21,29−33^ However, these MPCs have limitations for their use in aqueous biological applications due to their low biocompatibility and lack of ω-functional groups that allow interfacing with biomolecules. This problem critically hinders the use of MPCs as bioelectrocatalysts in biofuel cells or biosensors.

In the present work we report the synthesis of TA-AuMPCs showing discrete electron-charging behavior at ambient conditions in both organic and aqueous solutions, as well as for gold surface-anchored nanoclusters modified with different self-assembled monolayers (SAMs). Thioctic acid (TA) is an excellent capping ligand bearing a carboxylic termination which has been previously used for synthesis of gold nanoparticles and their further linkage to proteins,^34−37^ and for obtaining gold nanoclusters with luminescent properties.^38^

TA-AuMPCs were prepared following two methodologies based on the two-phase Brust-Schiffrin synthesis route:^39^ (i) ligand place-exchange by TA of hexanethiolate-coated MPCs (C6S-Au147) previously synthesized according to modifications described by Murray,^32,18,19^ and (ii) by using TA as the capping ligand at low temperatures in the presence of an excess of thiol, at a S:Au molar ratio of 1:1 (Supporting Information), and subsequently purified by column chromatography.^36^

The TA-AuMPCs obtained using both methods were characterized by UV–vis spectroscopy. Figure 1 shows the absorption spectra of the purified clusters. The characteristic surface plasmon band of Au nanoparticles (SPB) at 2.38 eV (520 nm) is not observed, indicating that the MPCs are smaller than 2 nm.^8,40^ Discrete electronic transitions are observed between 4 and 5 eV, and the fine structure of the spectra is more clearly visualized in the derivative spectra (Figure 1). The absorption bands are associated with interband transitions from the Au 5d^10^ to the unoccupied Au 6(sp)^1^ levels,^41^ although metal–ligand charge transfer can also contribute to the fine details of the spectra.^42^ FTIR spectroscopy showed the characteristic bands of TA molecules, confirming their presence as a constituent of the ligand shell (Figure S1, Supporting Information). High-resolution transmission electron microscopy (HR-TEM) indicated monodisperse clusters with an average diameter of (1.7 ± 0.2) nm (Figure S2).

**Figure 1:**
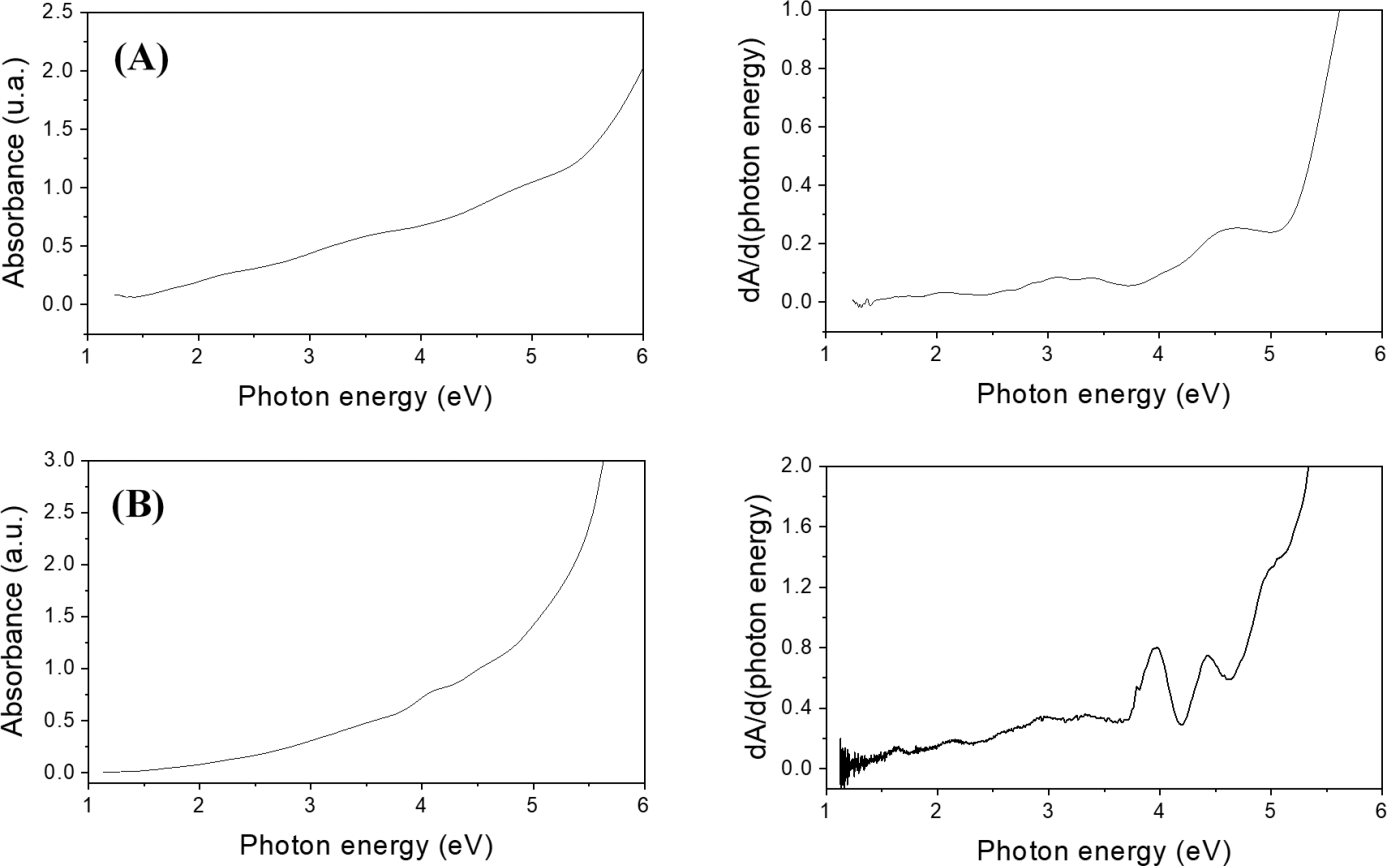
Absorption and derivative spectra of a solution of TA-AuMPCs in EtOH (A) and in Milli-Q water (B) obtained by procedure (i) or (ii), respectively.

Single-electron charging features of the clusters in solution were studied by differential pulse voltammetry (DPV). Figure 2 shows well-defined DPV quantized charging events of annealed C6MPCs in organic media, before and after ligand place-exchange by TA. The single-electron charging events of the MPCs are uniformly spaced with formal potentials described by
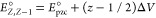
1where *E*°_Z,Z-1_ is the formal potential of the *z*/(*z* – 1) charge state couple. A linear dependence of *E*°_Z;Z-1_ on charge state (*Z*-plot) is observed (Figure 2) from which a capacitance value of *C*_MPC_= 0.59 aF was obtained for the C6-MPCs clusters, in agreement with that reported previously for C6S-Au147 clusters.^13−15,30^ This experimental value can be compared with a simple dielectric model involving a small spherical metallic core covered with a dielectric layer of thickness *d* and dielectric constant ϵ = 3 according to
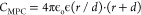
2where ϵ_o_ is the permittivity of free space (8.854 × 10^–12^ F m^–1^) and *r* is the metal core radius. For a core radius = 0.85 nm from TEM and a thickness of 0.77 nm for a C6 SAM,^14^ the theoretical value of CMPC agreed to the experimental one that was measured. DPV of TA-MPCs obtained after the ligand exchange reaction displayed a lower number of one-electron charging events and a *C*_MPC_ of 0.62 aF. Again, this value was equal to that theoretical one calculated for a layer thickness of 0.7 nm estimated for a thioctic acid SAM^43^ (Figure 2b). The MPCs were also immobilized on a 1,9-nonanedithiol self-assembled monolayer modified gold electrode. Anchoring was achieved by a ligand place-exchange reaction, in which one or more thiols on the SAM gold surface were replaced by thiolate ligands on the Au-MPCs.^44^Figure 2c shows the Coulomb staircase charging observed in organic media for TA-MPCs immobilized on a 1,9-nonanedithiol modified gold electrode in organic media. The DPV profile in CH_2_Cl_2_ showed a number of ET events, similar to those of freely diffusing MPCs in solutions but with a smaller potential separation. The *C*_MPC_ value determined from the slope of the linear *Z*-plot is 0.78 aF. This value is 25% larger than that observed when the particles were in solution, in agreement with that previously reported for alkanethiol Au-MPCs anchored onto an electrode surface.^30^ This result is in contrast to what is predicted in eq 2, where an increase of the effective thickness of the protecting monolayer (C9 vs C6) should lead to a smaller capacitance. However, it has been reported^45^ that the theoretical consideration based solely on the assumption of the MPC molecular capacitance is not sufficient to describe the redox behavior of surface self-assembled MPCs. In this case, the prediction is more complex since other factors, such as the effect of the electrostatic interaction between attached MPCs and the substrate electrode, besides the solvent dielectric should be considered.^46^

**Figure 2:**
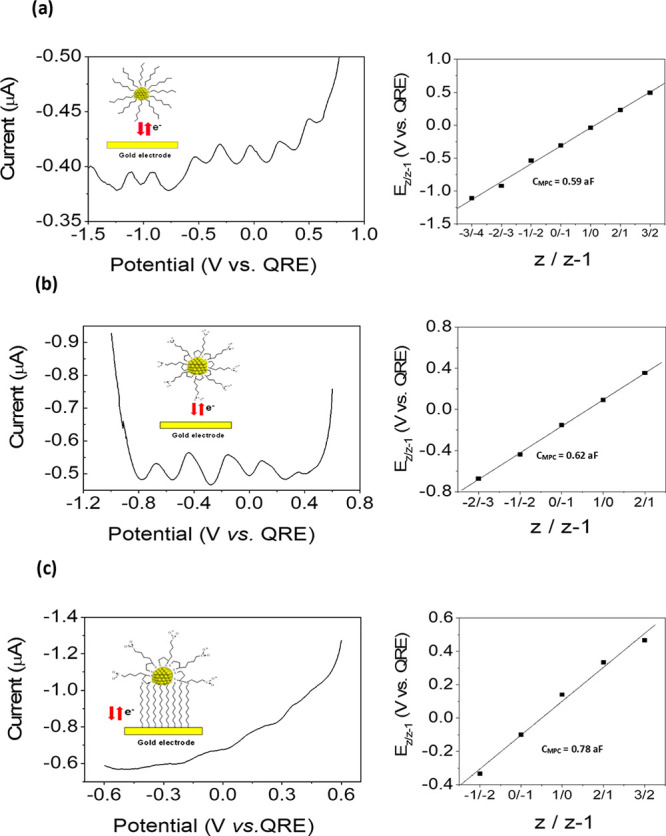
Differential pulse voltammograms (DPV) in organic media (CH_2_Cl_2_ + 0.1 M NBu_4_PF_6_ as supporting electrolyte) and plots of formal potentials of the quantized capacitance charging versus the valence states of: (a, b) C6-AuMPCs and TA-AuMPCs in solution; (c) TA AuMPCs immobilized on a 1,9-nonanedithiol self-assembled monolayer modified gold electrode. *C*_MPC_ is calculated from the Δ*V* according to *C*_MPC_ = *e*/slope of right plots, where *e* is the electron charge. QRE stands for Quasi Reference Electrode.

The capacitance properties of immobilized TA-MPCs in aqueous 0.1 M NH_4_PF_6_ were also investigated. Figure 3a shows their DPV response and the corresponding *Z*-plot, where linearity in the potential range of −0.1 to +0.6 V can be seen, as expected for QDL behavior. A value of *C*_MPC_ of 1.05 aF was estimated from these results, which is 36% larger than that obtained in organic solutions, in agreement with previous results obtained for other Au-MPCs.^30^ This increase is reported to be associated with the increment of dielectric constant (ϵ_d_) of the protecting monolayer of the MPC caused by the penetration electrolyte ions into SAM.^30^ It is also noteworthy that the quantized charging features are observed mainly at positive electrode potentials. It has been proposed that the anion PF_6_^–^ can induce the rectification of quantized capacitance charging at positive potentials. The rectification mechanism has been discussed by Chen et al.^30^ based on results obtained using the Randles’ equivalent circuit with *C*_SAM_ and *C*_EL_ as two capacitance constituents which account for the collective contributions of all surface-immobilized MPC molecules and the electrode surface defects, respectively. The incorporation of PF_6_^–^ anions at positive potentials can expel water molecules from the interfacial region.^30^ In consequence the measured capacitance is mainly due to charging through surface-anchored MPC molecules (*C*_SAM_). By contrast, at negative potentials, anion insertion is disfavored, and therefore, the interfacial charging is mainly through the electrode surface defects (*C*_EL_).^30^ This behavior is observed more clearly when TA-MPCs are immobilized on a SAM of 1,6-hexanedithiol (Figure 3b). In this case, the DPV results showed only positive QDL peaks with a *C*_MPC_ of 1.16 aF, which is larger than that obtained for nonanedithiol SAM due to the shorter chain length of 1,6-hexanedithiol.

**Figure 3:**
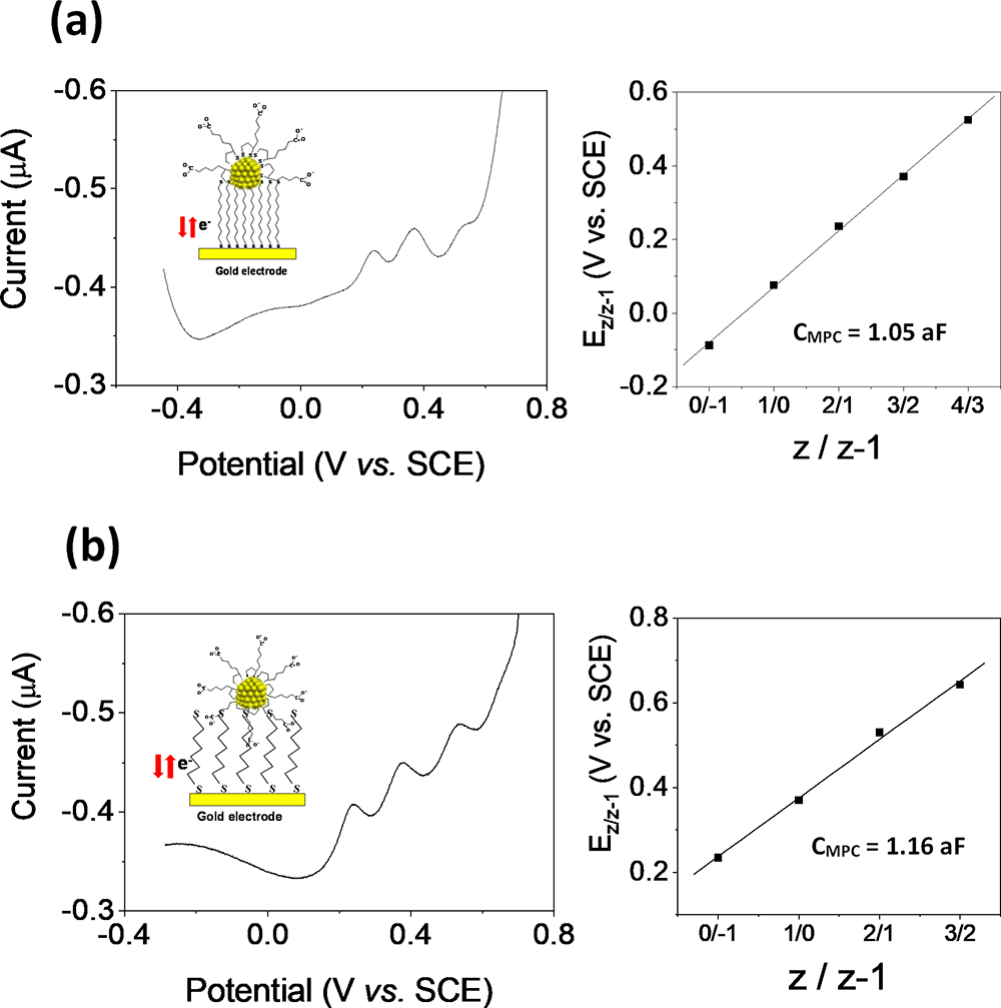
Differential pulse voltammograms (DPV) in aqueous media (NH_4_PF_6_) and the dependence of formal potentials of the quantized capacitance charging on the valence state of TA-AuMPCs immobilized on a: (a) 1,9-nonanedithiol; (b) 1,6-hexanedithiol self-assembled monolayer modified gold electrode.

In the case of TA-MPCs synthesized by the second methodology, the DPV in aqueous medium of gold clusters immobilized on nonanedithiol (Figure 4a) exhibited weak charging peaks, but in this case both at positive and negative potentials without ion-rectified quantized charging. This suggests that capping by TA molecules in these MPCs is more extensive than that obtained by the ligand place-exchange method, where not all hexanethiol molecules may have not been replaced. Thus, an increase of coverage by TA molecules would give rise to an increase of the negative charge of MPCs due to the carboxylic groups, preventing ion binding by the electrolyte anion (PF_6_^–^). In this way, a *C*_MPC_ of 1.18 aF was obtained, which is larger than the values reported above.

**Figure 4:**
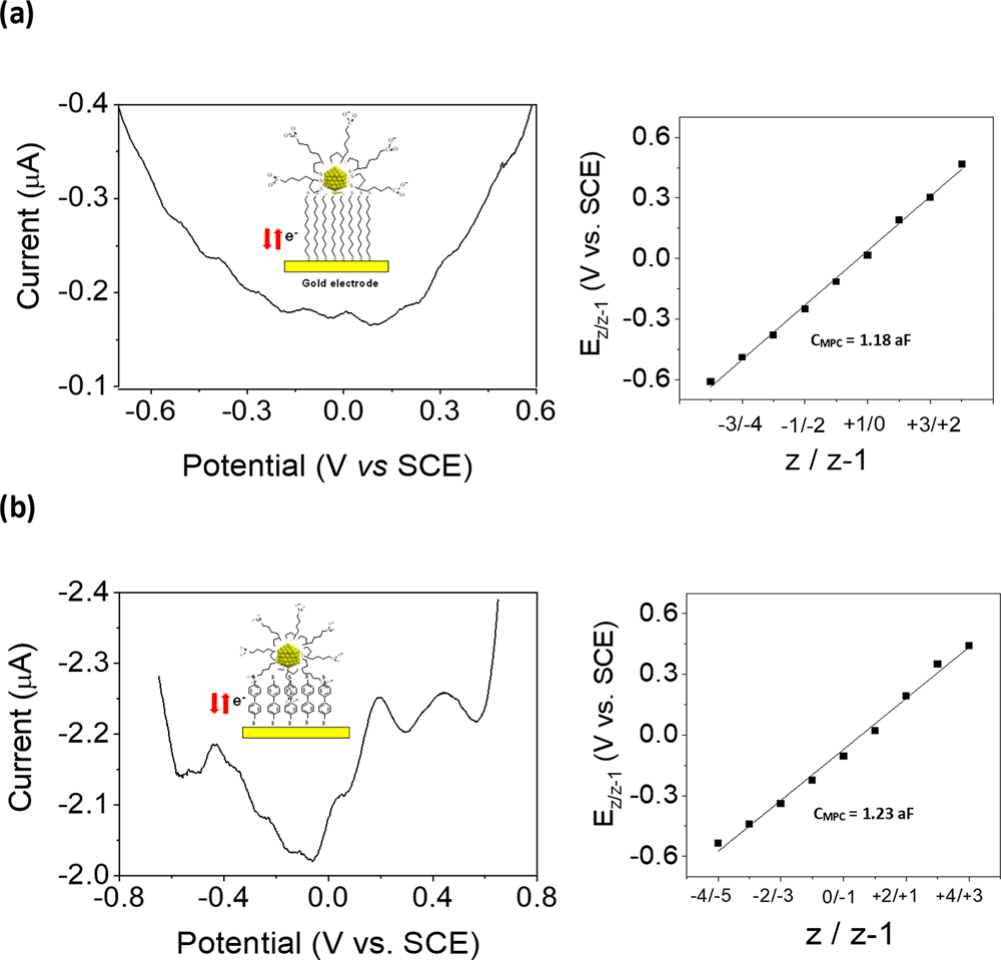
Differential pulse voltammograms (DPVs) in aqueous media using 0.1 M NH_4_PF_6_ as the supporting electrolyte and plots of formal potentials of the quantized capacitance charging versus their valence states of TA-AuMPCs immobilized on a: (a) 1,9-nonanedithiol; (b) biphenyl-4,4′-dithiol self-assembled monolayer modified gold electrode.

The effect of the linker on the MPC capacitance charging was also investigated by anchoring the TA-MPCs to a gold electrode functionalized with a SAM of biphenyl-4,4′-dithiol (Figure 4b). In this case, MPC electron transfer is expected to be favored with a higher conductivity bridge molecule and hence a lower charge-transfer resistance.^36^ Consequently, the DPV profiles exhibited larger and more distinct charging peaks both at positive and negative potentials, and a larger value of *C*_MPC_ of 1.23 aF was measured.

To summarize, we have reported the synthesis of gold clusters passivated by thioctic acid exhibiting single-electron charging features both in aqueous and organic media. Different capacitances were measured for TA-AuMPCs in solution and when anchored on self-assembled modified gold electrodes, which were dependent on the synthetic method employed. Ion-induced rectification was also observed in some cases. The TA-AuMPCs had long-term stability preserving their optical and electrochemical properties. This stability is associated with the larger lateral interactions between neighboring chains of the charged thioctic SAM improving the stability of the monolayer in comparison to that reported for *n*-alkanethiols SAMs.^47^ TA-AuMPCs offers a carboxylate termination, which is very convenient for further functionalization chemistry and allows their use in single-electron transfer processes in aqueous media for biological applications. Further research on this approach is currently ongoing.

## Supporting Information Available

Experimental section, FTIR characterization, and TEM images of TA-capped gold clusters (PDF)

## Abbreviations

TA: thioctic acid
AuMPCs: monolayer-protected gold clusters
QDL: quantized double layer
*C*_MPC_: capacitance monolayer protected cluster
DPV: differential pulse voltammetry
FTIR: Fourier-transform infrared spectroscopy
