# Supplementary material for: Single-Electron Charging of Thioctic Acid Monolayer-Protected Gold Clusters
Source: J Phys Chem Lett. 2023 Feb 3;14(6):1452–6. doi: 10.1021/acs.jpclett.2c03940 (PMC9940197; doi:10.1021/acs.jpclett.2c03940)
Supplement: Supplementary file 1 — jz2c03940_si_001.pdf [file jz2c03940_si_001.pdf]

Single-electron Charging of Thioctic Acid Monolayer-Protected Gold Clusters

Jose M. Abad, Marcos Pita, and Antonio L. De Lacey*

Instituto de Catálisis y Petroleoquímica, CSIC. C/Marie Curie 2, 28049 Madrid, Spain.

Experimental section

1. **Synthesis of the Thioctic Acid Monolayer protected gold clusters (TA-AuMPCs)**

The synthesis was carried out by two methodologies:

(i) **Ligand place-exchange by TA of hexanethiolate-coated MPCs (C_6S-Au_{140})**

Firstly, hexanethiolate-coated MPCs (C_6S-Au_{140}) were synthesized following procedures previously described[1,2] but with some modifications. To a vigorously stirred solution of 3 g of tetraoctylammonium bromide in 160 mL of toluene was added 0.72 g of $HAuCl_4$,·$3H_2O$ (Aldrich, 99.99%) in 50 mL of deionized water. The yellow $HAuCl_4$·$3H_2O$ aqueous solution quickly cleared and the toluene phase became orange-brown as the $AuCl_4^-$ was transferred into it. The organic phase was isolated and 796 µL of 1-hexanethiol (C6SH, 97%, Sigma-Aldrich) were added, and the resulting solution was stirred for 10 min at room temperature. The temperature was then adjusted at 0ºC and $NaBH_4$ solution (0.76 g in 50 mL) in cooled Milli-Q water was added. The reduction was allowed to proceed for 45 min after which the water layer was removed with a separatory funnel and the toluene solvent removed to a state of a moist black sludge using a rotary evaporator at temperatures ≤ 30ºC. A fraction (ca. 15%) of this material is soluble in EtOH and is extracted overnight by adding ca. 60 mL ethanol to the round-bottom flask. The product solution was filtered using a medium porosity number 4 glass fritted Buchner funnel rinsing the frit with ethanol. The ethanol was removed by rotary evaporation at room temperature and ca. 60 mL of acetonitrile was added to the solid which was allowed to stand overnight. The acetonitrile insoluble nanoparticles were collected using a glass fritted Buchner funnel and copiously rinsed with acetonitrile. The EtOH soluble C6-MPCs have an average core of 29 kDa of Au. This reaction produces[2,3] MPCs with an average composition of $Au_{140}[S(CH_2)_5CH_3]_{53}$ and an average 1.6 nm core diameter.

The MPCs were annealed by co-dissolving 40 mg of EtOH-soluble C6 MPCs in 8.5 mL CH_2Cl_2 and adding 1.65 µL of 1-hexanethiol (1:5.3 molar ratio, of C6SH relative to hexanethiolate ligands on the MPC) and allowed to stand at room temperature for 96 h. The solvent was removed with rotary evaporation (without heating) and 40 mL of acetonitrile was added to the resulting solid sample and sonicated for ~5 min to dissolve residual thiol. The annealed C6 MPC solid was allowed to settle (it is insoluble in

acetonitrile), and the solvent was decanted by filtration with a porosity glass (number 4) fritted Buchner funnel. The MPCs collected were rinsed with acetonitrile an additional two times and allowed to air-dry.

Ligand exchange reaction[3] using Thioctic acid (TA, 1,2-dithiolane-3-pentanoic acid; Sigma-Aldrich) was carried out employing a 3:1 molar ratio of TA relative to C6 ligand on the MPC. 30 mg of the C6-MPCs annealed were dissolved in 5 mL of CH_2Cl_2 and TA solution (1.4 mL, containing 0.029 grams in CH_2Cl_2) was added. The mixture was stirred for four days at room temperature. Following, the solvent was removed with rotary evaporation (without heating) and several aliquots of acetonitrile were added to the resulting solid sample and sonicated for ~5 min to remove residual TA. The product was filtrated by a porosity glass (number 4) fritted Buchner funnel and washed in the filter with acetonitrile to remove any TA residue and allowed to air-dry.

(ii) Synthesis of TA-AuMPCs using TA as the capping ligand

The clusters were prepared by the two-phase method of Brust et al.[4,5] Typically, $AuCl_4^-$ was transferred from an aqueous solution (6 mL, 30 mM, HAuCl4·3H_2O, Aldrich, 99.99%) to the organic phase using tetraoctylammonium bromide (TOABr, Sigma-Aldrich) in toluene as the phase-transfer reagent (16 mL, 50 mM, Sigma-Aldrich) with vigorous stirring for 30 min. The aqueous phase was discarded. Thioctic acid (TA, 1,2-dithiolane-3-pentanoic acid) (2 mL, 90 mM in toluene, Sigma-Aldrich) was then added and stirred for another 30 min. A freshly prepared cooled aqueous solution of sodium borohydride (5 mL, 0.4 M, Sigma-Aldrich) was rapidly added to the solution mixture at ~3 °C under vigorous stirring. The organic layer turned cloudy brown, and the mixture was left stirring for 3 h at ~3 °C. The clusters formed after reduction were soluble in the aqueous phase due to their carboxylate termination, and the organic phase was discarded. They were purified and separated through a chromatographic column of Sephadex G-25 previously equilibrated and eluted using milli-Q water slightly alkaline.

2. Gold electrode preparation

Polycrystalline gold disk electrodes (0.5 cm in diameter, 0.196 cm^2) were polished with 1 μm alumina powder (Buehler GmbH, Germany), rinsed, and sonicated for 15 min in Milli-Q water. The electrodes were then dipped in 0.1 M H_2SO_4 and activated by holding the potential at +2.0 V for 5 s and then at −0.35 V for 10 s, followed by potential cycling from −0.35 to +1.5 V at 4 V/s for 100 scans. Finally, the CV characteristic of a clean polycrystalline gold surface was recorded in 0.1 M H_2SO_4 at 0.1 V/s. The microscopic area was calculated by integration of the cathodic peak associated with the reduction of the gold oxide using a value of 482 $\mu C \cdot cm^{-2}$ for a monolayer of chemisorbed oxide on polycrystalline gold.[6] After the cleaning procedure, the gold electrodes were immersed for 24 h in a 1 mM solution of respective dithiols: 1,9-nonanedithiol; 1,6-hexanedithiol; biphenyl-4,4′-dithiol (Aldrich) in ethanol. The electrodes were subsequently rinsed with ethanol and dried in air. Attachment of the TA-AuMPCs onto the modified dithiol gold electrode was carried out by incubation of the electrode for 24 h in a 40 μM (concentration of clusters) colloidal solution of TA-AuMPCs obtained from the synthesis described above. The electrodes were subsequently washed with Milli-Q water.

3. Electrochemical measurements

Differential pulse voltammetry data were obtained with an Autolab potentiostat (PGSTAT 30, Eco Chemie) using a three-electrode cell inside a Faraday cage shielding from environmental electronic noise, allowing for high quality electrochemical measurements. Platinum gauze was used as counter electrode, and the potentials were measured with respect to a saturated calomel electrode (SCE) and a silver wire as a quasi-reference electrode (QRE) in aqueous and organic medium, respectively. All solutions were

deoxygenated by bubbling nitrogen before the measurements, and all experiments were carried out at room temperature, 22 ± 2 °C. Ammonium hexafluorophosphate (0.1M, NH_4PF_6; Sigma-Aldrich) and tetrabutylammonium hexafluorophosphate (0.1M, NBu_4PF_6; Sigma-Aldrich) were used as supporting electrolytes in aqueous and organic medium, respectively. The optimized instrumental parameters of DPV were as follows: Modulation amplitude (mV) = 20; Modulation time (ms) = 20; Interval time (ms) = 200; deposit time (s) = 5; quiet time (s) = 5.

4. FTIR spectroscopy measurements

TA-MPCs were dissolved in CH_2Cl_2 and a drop was evaporated to dryness on a polished CaF_2 window. The IR spectra were recorded in a Tensor Bruker Fourier transform spectrometer equipped with an MCT detector and a purge gas system for removal of CO_2 and H_2O (Whatman). The IR spectra were averaged over 124 scans; the spectral resolution was 2 cm^{-1}. The spectra were blank subtracted and baseline corrected.

5. Transmission electron microscopy (HR-TEM)

The morphology and size distribution of the nanoparticles were measured with a 200 KV JEOL 2100 TEM/STEM microscope. Coupled Energy-dispersive X-ray analyzer (EDX) from Oxford Instruments was used for the elemental surface analysis. The samples for examination by HR-TEM were prepared by evaporation of a drop of nanoparticle solution on carbon films supported on standard copper grids. Mean particle size and standard deviation were determined from measurements of at least 200 particles.

6. Supporting figures

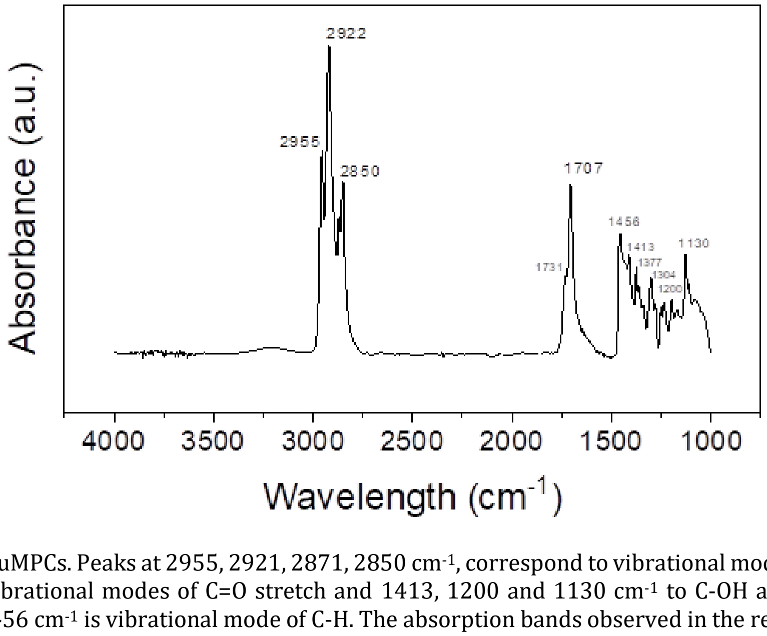

Figure S1. FTIR spectrum of TA-AuMPCs. Peaks at 2955, 2921, 2871, 2850 cm^{-1}, correspond to vibrational modes of $\nu_{asy}(-CH_2)$, $\nu_{sym}(-CH_2)$; 1731 and 1707 cm-1 are vibrational modes of C=O stretch and 1413, 1200 and 1130 cm^{-1} to C-OH and C-O stretch due to carboxylic group of TA. Band at 1456 cm^{-1} is vibrational mode of C-H. The absorption bands observed in the region 1400–1200 cm^{-1} are associated to the deformation vibrations of the C–H bonds in hydroxyl group and bands in the region 1200–1000 cm^{-1} are valence vibrations of the C–O bonds. Nanoparticles were drop-cast on a CaF_2 window and left to dry.

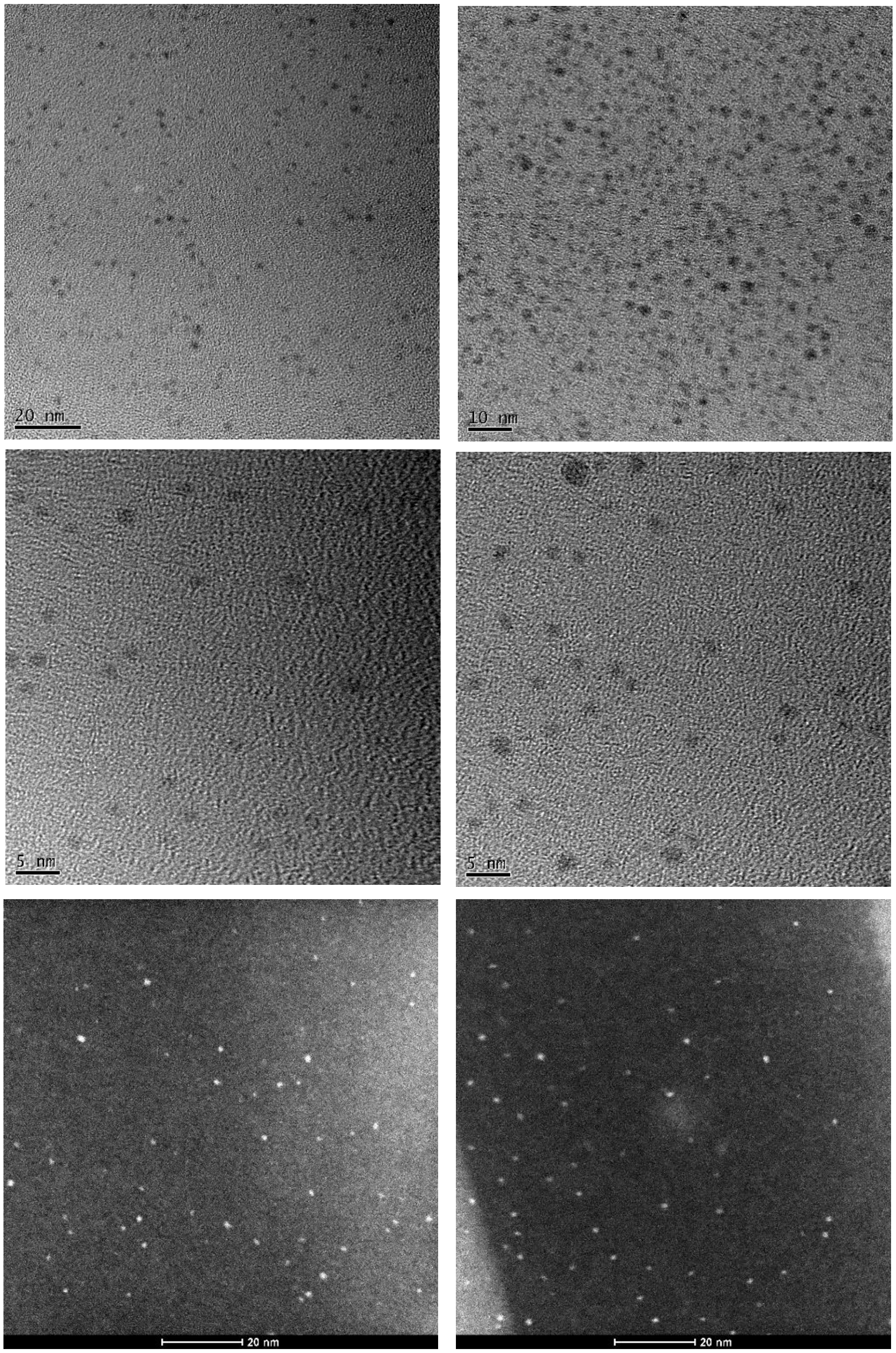

Figure S2. TEM images of TA-derivatized gold clusters.

References

(1) Hostetler, M. J.; Stokes, J. J.; Murray, R. W. Infrared Spectroscopy of Three-Dimensional Self-Assembled Monolayers: N-Alkanethiolate Monolayers on Gold Cluster Compounds. *Langmuir* **1996**, *12*, 3604–3612.

(2) Hostetler, M. J.; Wingate, J. E.; Zhong, C.-J.; Harris, J. E.; Vachet, R. W.; Clark, M. R.; Londono, J. D.; Green, S. J.; Stokes, J. J.; Wignall, G. D.; Glish, G. L.; Porter, M. D.; Evans, N. D.; Murray, R. W. Alkanethiolate Gold Cluster Molecules with Core Diameters from 1.5 to 5.2 nm: Core and Monolayer Properties as a Function of Core Size. *Langmuir* **1998**, *14*, 17–30.

(3) Miles, D. T.; Murray, R. W. Temperature-Dependent Quantized Double Layer Charging of Monolayer-Protected Gold Clusters. *Anal. Chem.* **2003**, *75*, 1251–1257.

(4) Brust, M.; Walker, M.; Bethell, D.; Schiffrin, D. J.; Whyman, R. Synthesis of Thiol-derivatised Gold Nanoparticles in a Two-phase Liquid–Liquid System. *J. Chem. Soc. Chem. Comm.* **1994**, 801–802.

(5) Abad J.M.; Gass, M.; Bleloch, A; Schiffrin, D. J. Direct Electron Transfer to a Metalloenzyme Redox Center Coordinated to a Monolayer-Protected Cluster. *J. Am. Chem. Soc.* **2009**, *131*, 10229–10236.

(6) Oesch, U.; Janata, J. Electrochemical Study of Gold Electrodes with Anodic Oxide Films—I. Formation and Reduction Behaviour of Anodic Oxides on Gold. *Electrochim. Acta* **1983**, *28*, 1237–1246.
